# Hsp90 as a pathophysiological factor and emerging therapeutic target in atopic dermatitis

**DOI:** 10.3389/fimmu.2025.1658399

**Published:** 2025-08-28

**Authors:** Stefan Tukaj

**Affiliations:** Laboratory of Cellular and Molecular Immunology, Department of Molecular Biology, Faculty of Biology, University of Gdansk, Gdansk, Poland

**Keywords:** molecular chaperones, inflammation, NF-κB, STAT, eosinophils, skin barrier, microbiome, *Staphylococcus aureus*

## Abstract

Heat shock protein 90 (Hsp90) is a molecular chaperone that plays a critical role in stabilizing and regulating numerous client proteins involved in inflammation, immune activation, and skin barrier homeostasis. Emerging evidence suggests that Hsp90 contributes to the pathophysiology of atopic dermatitis (AD), a chronic inflammatory skin disorder characterized by immune dysregulation, epidermal barrier dysfunction, and microbial imbalance. Notably, elevated intracellular Hsp90 activity has been reported in peripheral blood leukocytes of AD patients, alongside increased extracellular Hsp90 and anti-Hsp90 IgE antibodies. Preclinical studies employing murine models of AD, including dinitrochlorobenzene (DNCB)- and calcipotriol (MC903)-induced dermatitis, have demonstrated that both topical and systemic inhibition of Hsp90 ameliorates disease severity. These improvements correlate with reduced epidermal hyperplasia, decreased expression of Th/Th2 cytokines, attenuation of keratinocyte-derived alarmins, and suppression of inflammation. Additionally, Hsp90 inhibition limits the infiltration or activation of immune cells such as T cells, neutrophils, eosinophiles, and mast cells in the skin. Mechanistic investigations reveal that Hsp90 blockade downregulates key signaling pathways implicated in AD pathogenesis, notably NF-κB and JAK-STAT. *In vitro* studies further corroborate that Hsp90 inhibition reduces proinflammatory responses in keratinocytes, CD4^+^ T cells, and eosinophils. Beyond modulating skin inflammation, Hsp90 blockade partially restores gut microbiota dysbiosis and impairs *Staphylococcus aureus* biofilm formation, both relevant to AD pathogenesis. Although clinical data on Hsp90 inhibitors in AD are still lacking, early-phase trials in psoriasis and hidradenitis suppurativa suggest potential therapeutic benefit. Collectively, these findings underscore a multifaceted role for Hsp90 in AD and support its potential as a promising novel therapeutic target.

## Introduction

Atopic dermatitis (AD) is a highly prevalent chronic inflammatory skin disease characterized by relapsing eczematous lesions, pruritus, and profound impact on quality of life. Although recent therapeutic developments have improved disease management, AD remains incurable for most patients, with a high burden of disease persistence and relapse. Its multifactorial pathogenesis—encompassing genetic predisposition, environmental triggers, epidermal barrier dysfunction, and immune dysregulation—poses substantial diagnostic and therapeutic challenges (1–4). Recent advances in immunological research have expanded our understanding AD beyond the traditional Th2-dominated paradigm. AD exhibits a complex immune landscape, including significant involvement of Th22, Th1, and Th17 pathways depending on the subtype of the disease. This heterogeneity is further supported by single-cell transcriptomic studies and biomarker-based endotyping, which reveal distinct immune signatures and anatomical site–specific inflammation, underscoring the need for personalized therapeutic approaches targeting multiple immune axes (5, 6). Simultaneously, the role of the microbiome has gained prominence: *Staphylococcus aureus* colonization and microbial imbalance correlate with barrier defects and disease severity, while gut–skin axis perturbations may influence systemic immune priming (7). Environmental factors, including urbanization, pollution, and climate variability, further contribute to AD exacerbations and chronicity. The exposome framework is increasingly used to interpret environmental contributions to AD, recognizing the cumulative effect of chemical, physical, and psychosocial exposures throughout life (8). Despite the advent of targeted systemic therapies—such as biologics directed against IL - 4Rα, IL - 13, IL - 31 and small-molecule JAK inhibitors—a large subset of patients do not achieve long-term remission. Primary non-response, secondary loss of efficacy, and treatment discontinuation remain common (9, 10). These limitations underscore an urgent need to identify and therapeutically target upstream pathological mechanisms that drive chronicity, immune imbalance, and barrier dysfunction in AD. Among emerging intracellular regulators, heat shock protein 90 (Hsp90) has gained increasing attention as a therapeutic target, with growing evidence from *in vitro* studies, preclinical models, and a phase I clinical proof-of-concept trial in patients with plaque-type psoriasis (11, 12). The aim of this mini-review is to provide a focused analysis of the role of Hsp90 in the pathophysiology of atopic dermatitis and to evaluate the therapeutic potential of its inhibitors as a novel class of disease-modifying agents.

## HSP90 as a master regulator of cellular signaling and immune responses

Heat shock proteins (HSPs) are a highly conserved family of molecular chaperones that preserve cellular proteostasis by assisting in the folding, assembly, stabilization, and refolding of client proteins. Among them, heat shock protein 90 (HSP90) is one of the most abundant and versatile, comprising up to 2% of total cellular protein under basal conditions and increasing further under stress. HSP90 plays a crucial role in maintaining protein homeostasis and mediating various signal transduction pathways in both physiological and pathological contexts. The HSP90 family consists of multiple isoforms, including cytosolic HSP90α (HSP90AA1, inducible) and HSP90β (HSP90AB1, constitutive), GRP94/gp96 located in the endoplasmic reticulum, and TRAP1 in mitochondria (13). Each HSP90 homolog performs distinct roles tailored to its cellular localization. HSP90AA1 (HSP90α), inducible under stress, predominantly assists in folding and stabilizing client proteins involved in signal transduction and stress responses in the cytosol. In contrast, HSP90AB1 (HSP90β) maintains essential proteostasis during normal conditions, supporting constitutive cellular functions. GRP94, residing in the endoplasmic reticulum, is a pivotal component of the unfolded protein response (UPR), ensuring protein quality control and assisting in the maturation of secretory and membrane proteins. Meanwhile, TRAP1 functions within mitochondria to regulate bioenergetics, modulate reactive oxygen species (ROS) levels, and inhibit apoptosis, thereby contributing to cellular survival. These compartment-specific activities have been predominantly explored within oncology, revealing their contributions to cancer cell metabolism, proliferation, and resistance to stress. Crucially, the intracellular functions of these homologs are functionally and mechanistically distinct from their extracellular roles, underscoring the complexity of HSP90 biology in health and disease (14).

HSP90 operates through a dynamic ATPase cycle governed by its N-terminal, middle, and C-terminal domains. This cycle is tightly regulated by post-translational modifications (PTMs) such as acetylation, phosphorylation, and SUMOylation, which modulate HSP90’s interaction with co-chaperones and its repertoire of client proteins (15, 16). Functionally, HSP90 stabilizes a broad range of client proteins, including kinases (e.g., JAK), nuclear hormone receptors, and transcription factors. Many of these proteins are central to signaling pathways that control proliferation, differentiation, immunity, and survival. Key cascades supported by HSP90 include JAK/STAT, NF-κB, MAPK/ERK, PI3K/AKT, and TLR signaling. By facilitating the proper folding and function of these proteins, HSP90 orchestrates critical cellular responses to environmental stimuli (17). Importantly, HSP90 is also found outside cells. Extracellular HSP90 (eHSP90), particularly the α isoform, can be actively secreted in response to stress or hypoxia, or passively released from necrotic or damaged cells. eHSP90 functions as a damage-associated molecular pattern (DAMP) and engages with pattern recognition receptors such as Toll-like receptor 4 (TLR4), CD91, and LOX - 1 to modulate immune and inflammatory signaling. These interactions activate downstream NF-κB and MAPK cascades, increasing the production of pro-inflammatory cytokines, chemokines, and matrix metalloproteinases (MMPs), and facilitating leukocyte recruitment, tissue remodeling, and cell motility (18, 19). Beyond HSP90α, other HSP90 homologs also contribute to the extracellular signaling landscape. GRP94 and HSP90β have been detected in secretomes of stressed or transformed cells, where they can influence antigen presentation, modulate macrophage and dendritic cell activation, and participate in the regulation of innate immune checkpoints. Extracellular GRP94, in particular, has been implicated in chaperoning immunogenic peptides and facilitating their transfer to antigen-presenting cells, thereby linking cellular stress to adaptive immune responses. TRAP1, although predominantly mitochondrial, may also exert extracellular immunoregulatory effects upon non-canonical release, though the mechanisms remain less well defined. Collectively, these extracellular activities of HSP90 isoforms expand their canonical intracellular functions and position them as key mediators of intercellular communication in immunity and disease contexts (14).

Beyond its roles in immunity and inflammation, HSP90 is a critical enabler of oncogenic transformation. It stabilizes a range of oncogenic client proteins, including mutated p53, BCR-ABL, HER2, and others, thereby supporting tumor growth, metastasis, and resistance to therapy. As a result, HSP90 inhibitors such as geldanamycin derivatives (e.g., 17-AAG) and newer small molecules have been developed to disrupt these interactions. By promoting proteasomal degradation of oncogenic proteins, HSP90 inhibitors exhibit potent anti-cancer activity. Several such compounds are in clinical trials across various malignancies, highlighting the translational promise of targeting HSP90 in oncology (13, 17).

Given the central involvement of HSP90 in cellular signaling, immune regulation, and oncogenesis—both inside and outside the cell—this chaperone remains a critical target for therapeutic intervention. A detailed understanding of its molecular structure, regulation, and diverse biological functions forms the foundation for exploring its roles in human disease and for developing targeted strategies to modulate its activity.

## Hsp90 as a potential therapeutic target in autoimmune bullous skin diseases

Although this review focuses primarily on AD, it is important to acknowledge the roles and therapeutic potential of Hsp90 in other inflammatory and autoimmune skin disorders. Certain signaling pathways and pathophysiological mechanisms, while disease-specific, share common elements across conditions such as autoimmune bullous dermatoses, where Hsp90 has also emerged as a promising target. Autoimmune bullous skin diseases (AIBDs), such as bullous pemphigoid (BP), pemphigus vulgaris (PV), and epidermolysis bullosa acquisita (EBA), represent a group of rare, chronic, and potentially life-threatening disorders characterized by autoantibody-mediated blister formation at different levels of the skin. These diseases involve autoantibodies targeting structural proteins of the skin, resulting in loss of cell adhesion and subsequent blistering. Given the pathogenic role of autoantibodies and immune cells, targeting immune mechanisms has been central to therapeutic development. Recently, the molecular chaperone heat shock protein 90 (Hsp90) has emerged as a promising target in AIBDs due to its multifaceted roles in immune regulation and inflammatory signaling (12).

Initial evidence for the involvement of Hsp90 in AIBDs was demonstrated in EBA, an AIBD characterized by autoantibodies targeting type VII collagen. The administration of two Hsp90 inhibitors, 17-DMAG and TCBL - 145, in a mouse model of experimental EBA produced significant therapeutic effects. Both inhibitors markedly improved clinical signs of disease, as evidenced by reduced skin blistering and inflammation. Additionally, treatment suppressed the production of pathogenic autoantibodies against type VII collagen and diminished dermal infiltration of neutrophils, a key driver of tissue damage in EBA. Importantly, these beneficial effects were achieved without reducing the overall numbers of plasma cells, including those specific for type VII collagen, or germinal center B cells. Instead, the inhibitors exerted their immunomodulatory effects primarily through potent inhibition of T cell proliferation, demonstrated by decreased responsiveness of lymph node T cells to restimulation *in vitro* with anti-CD3/CD28 antibodies or autoantigen. These results indicate that Hsp90 blockade selectively targets pathogenic T cell responses while sparing plasma cells, offering a focused approach to modulating T-cell mediated autoimmunity in EBA (20).

Extending these findings to the skin microenvironment, topical application of the Hsp90 inhibitor 17AAG significantly ameliorated disease severity in both passive transfer and active immunization mouse models of EBA. This treatment reduced neutrophilic infiltration, inhibited NF-κB activation, downregulated MMPs and Flii (an actin-remodeling protein), and induced anti-inflammatory Hsp70 expression in the skin. The topical route offers the advantage of localized inhibition, minimizing systemic toxicity while effectively modulating key inflammatory pathways involved in autoantibody-mediated skin inflammation (21).

In another study, 17-DMAG dose-dependently reduced dermal–epidermal separation ex vivo and inhibited neutrophil-derived ROS production in response to fMLP, LPS, and EBA immune complexes. Additionally, extracellular Hsp90 was found to interact with MMP - 2 and MMP - 12 in EBA patient sera, suggesting their dependence on its chaperone function (22).

In bullous pemphigoid (BP), the most common subepidermal autoimmune blistering disease, Hsp90 expression was found to be dysregulated. Although intracellular Hsp90 levels were increased in perilesional skin biopsies from BP patients, circulating serum levels of Hsp90 were decreased and inversely correlated with IgG autoantibody titers against the immunodominant NC16A domain of BP180, a hemidesmosomal protein targeted by pathogenic autoantibodies. This suggests an abnormal regulation of intracellular and extracellular Hsp90 in BP pathogenesis. Importantly, Hsp90 was induced in keratinocytes by BP patient sera and purified anti-BP180 autoantibodies but was secreted in a restricted manner, further implicating this chaperone in the local skin inflammatory milieu (23).

Mechanistically, Hsp90 modulates keratinocyte responses to autoantibodies. *In vitro* studies demonstrated that Hsp90 inhibition with 17-DMAG suppressed IL - 8 production by keratinocytes stimulated with BP IgG, while IL - 6 secretion remained unaffected. This effect was mediated by the inhibition of NF-κB transcriptional activity and concomitant induction of Hsp70, a known negative regulator of inflammation. Since IL - 8 plays a critical role in neutrophil recruitment and activation in BP lesions, Hsp90 blockade interferes with a key pathogenic axis, thereby reducing local inflammation and tissue damage (24).

Humoral immune responses are also modulated by Hsp90 inhibition. *In vitro* treatment of activated human B cells with the Hsp90 blocker 17-DMAG reduced proliferation and IgG secretion, accompanied by induction of heat shock factor 1 (HSF1) and Hsp70, which may contribute to immunoregulatory feedback. *In vivo*, early treatment of type VII collagen-immunized mice with 17-DMAG altered splenic B cell subsets, increasing regulatory B cell fractions and serum IL - 10 levels while reducing circulating autoantibodies and disease severity. These findings illustrate that Hsp90 inhibition impacts both cellular and humoral arms of autoimmunity, supporting its therapeutic potential in antibody-mediated skin diseases (25).

Lastly, elevated circulating autoantibodies against Hsp90 have been detected in patients with dermatitis herpetiformis (DH), a blistering cutaneous manifestation of celiac disease, but not in BP or PV patients. In DH, levels of anti-Hsp90 autoantibodies correlated positively with disease activity and decreased during remission following gluten-free diet, suggesting a disease-specific autoimmune response against Hsp90 that may contribute to pathogenesis or serve as a biomarker (26).

Collectively, these studies provide converging evidence that Hsp90 plays critical roles in the pathophysiology of autoimmune bullous skin diseases by supporting pathogenic autoantibody production, neutrophil effector functions, and keratinocyte inflammatory responses. Pharmacologic inhibition of Hsp90 modulates these processes at multiple levels, offering a promising therapeutic strategy with potential for both systemic and topical application.

The findings outlined above establish Hsp90 as a critical mediator of autoimmune inflammation in the skin. Although AIBDs and AD differ in their primary immune pathways, they share important downstream mechanisms, including T cell-driven inflammation, keratinocyte activation, neutrophil infiltration, and cytokine-driven tissue damage. These shared effector pathways are regulated in part by Hsp90, as demonstrated in both *in vitro* and *in vivo* studies. Therefore, the role of Hsp90 in AIBDs provides valuable precedent and mechanistic rationale for its exploration as a therapeutic target in AD. By demonstrating how Hsp90 modulates immune activation, tissue inflammation, and skin-specific immune responses in AIBDs, this section supports the broader concept of Hsp90 as a central regulator of cutaneous immunopathology - setting the stage for the subsequent focus on its role and inhibition in AD (**Table 1**).

**Table 1:** Hsp90 as a potential therapeutic target in autoimmune/inflammatory skin diseases.

Model/System	Treatment/Intervention	Findings/Observations	Mechanism/Biological effects	Ref.
Mouse model (experimental EBA)	17-DMAG, TCBL-145, systemic	Reduced skin blistering and inflammation; decreased anti-collagen VII autoantibodies and dermal neutrophil infiltration	Selective inhibition of T cell proliferation; spared plasma cells and GC B cells	(20)
Mouse model (EBA, active and passive)	17-AAG, topical	Reduced disease severity, neutrophilic infiltration; inhibited NF-κB, downregulated MMPs and Flii; induced Hsp70	Local inhibition of inflammatory mediators; minimized systemic toxicity	(21)
Ex vivo (human skin and neutrophils)	17-DMAG	Reduced dermal-epidermal separation; inhibited neutrophil-derived ROS; extracellular Hsp90 interacted with MMP-2 and MMP-12	Hsp90 supports MMP activity and neutrophil activation in EBA	(22)
BP patients (skin and serum)	None	Increased intracellular Hsp90 in skin; decreased serum Hsp90 levels inversely correlated with anti-BP180 IgG	Dysregulated intra-/extracellular Hsp90 in BP	(23)
*In vitro* (keratinocytes stimulated with BP IgG)	17-DMAG	Reduced IL-8 but not IL-6; inhibited NF-κB and induced Hsp70	Suppression of IL-8-dependent inflammation	(24)
*In vitro* (human B cells); mouse model (EBA)	17-DMAG	Reduced IgG secretion and B cell proliferation; increased IL-10 and regulatory B cells; decreased disease severity	HSF1/Hsp70-mediated feedback; modulated humoral and regulatory immune responses	(25)
DH patients	None	Elevated anti-Hsp90 autoantibodies correlated with disease activity; decreased with gluten-free diet	Disease-specific Hsp90 autoimmunity; potential biomarker	(26)
AD patients (serum)	None	Elevated serum Hsp90 and anti-Hsp90 IgE autoantibodies; correlation with AD severity (SCORAD)	Extracellular Hsp90 and IgE autoantibodies may contribute to AD pathogenesis	(27)
AD patients (serum)	None	Elevated circulating Hsp90α in AD compared to DH, CD, and healthy controls	Disease-specific dysregulation of extracellular Hsp90α	(28)
Mouse model (DNCB-induced AD)	STA-9090 (Ganetespib), systemic/topical	Reduced clinical severity, epidermal thickening, leukocyte infiltration, scratching; restored filaggrin expression	Hsp90 inhibition modulates immune dysregulation and barrier function	(29)
*In vitro* (keratinocytes) + MC903-induced AD in mice	RGRN-305, oral and topical	Downregulation of Th1/Th2 cytokines and chemokines; reduced skin inflammation and leukocyte infiltration	Inhibition of STAT3 and STAT6 activation; reversal of inflammatory gene signature	(30)
Mouse model (DNCB-induced AD) + human samples	17-AAG, topical	Reduced dermatitis scores, epidermal hyperplasia, TSLP, IL-5, IL-6; suppressed eosinophil and mast cell activity; partial microbiome restoration	Inhibition of NF-κB; decreased cytokine production; decreased ROS; altered Hsp90 acetylation; inhibited S. aureus biofilm formation	(31)
*In vitro* and *in vivo* (contact hypersensitivity/AD-like)	Geldanamycin or Hsp90-knockout mice	Suppressed immune activation and contact hypersensitivity responses	DNCB-modified Hsp90 activates immune cells via Hsp90’s receptor CD91	(32)
Patients with plaque-type psoriasis	RGRN-305, oral	Marked clinical improvement in a subset of patients with moderate-to-severe psoriasis; normalization of key inflammatory pathways including TNF-α, IL-23, and IL-17A signaling	Hsp90 inhibition modulates major inflammatory signaling pathways in psoriasis	(11)
Patients with hidradenitis suppurativa (HS)	RGRN-305, oral	Clinically meaningful efficacy with over half of treated patients achieving HiSCR-50 responses; favorable safety profile, no serious adverse events	Hsp90 inhibition reduces inflammation and symptoms in HS	(34)

## Hsp90 as a potential therapeutic target in atopic dermatitis

Atopic dermatitis (AD) remains a challenging chronic inflammatory skin disorder characterized by a complex interplay of immune dysregulation, epidermal barrier dysfunction, and altered skin microbiome. Despite advances in understanding its pathophysiology, new therapeutic strategies are continuously sought to improve disease control and patient outcomes. Among emerging targets, heat shock protein 90 (Hsp90), a highly conserved molecular chaperone, has attracted increasing attention due to its involvement in regulating multiple signaling pathways relevant to inflammation and immune responses. Experimental data accumulated over recent years highlight the potential of Hsp90 inhibition as a novel therapeutic approach in AD, supported by *in vitro*, *in vivo*, and translational studies.

Initial explorations into the role of Hsp90 in skin inflammation and autoimmunity underscored its importance in maintaining the stability and function of numerous client proteins, including kinases and transcription factors pivotal in immune signaling cascades. AD is a chronic inflammatory skin disorder associated with IgE-mediated immune dysregulation. The first study suggesting a potential role of heat shock protein 90 (Hsp90) in the pathogenesis of AD revealed a significant elevation of serum Hsp90 levels and anti-Hsp90 IgE autoantibodies in AD patients compared to healthy controls. Notably, approximately 50% of AD patients exhibited seropositivity for anti-Hsp90 IgE, in contrast to less than 3% of healthy individuals. A positive correlation between serum Hsp90 levels and disease severity, as measured by SCORAD, was also demonstrated. These findings indicate that extracellular Hsp90 and IgE autoantibodies targeting Hsp90 may contribute to the immunopathology of AD, supporting the hypothesis of an autoimmune component in the disease (27).

Extending this concept, further studies demonstrated that circulating Hsp90α - a major extracellular isoform - was markedly elevated in AD patients when compared not only to healthy controls but also to individuals with dermatitis herpetiformis (DH) and celiac disease (CD). While serum Hsp90α levels remained comparable across DH, CD, and healthy groups, AD patients showed a distinct overexpression, suggesting disease-specific dysregulation of this chaperone. These findings indicate that extracellular Hsp90α may not only contribute to the inflammatory processes in AD but could also aid in distinguishing AD from other pruritic or autoimmune dermatoses such as DH. However, the diagnostic utility of Hsp90α remains preliminary and warrants further investigation (28).

Building on the emerging role of extracellular Hsp90 in AD, recent preclinical findings provided functional evidence that pharmacological inhibition of this chaperone may hold therapeutic promise. For the first time, the effects of the Hsp90 inhibitor STA - 9090 (Ganetespib) were evaluated in an experimental mouse model of dinitrochlorobenzene (DNCB)-induced AD. Both systemic (intraperitoneal) and topical administration of STA - 9090 significantly alleviated disease manifestations. Treated animals exhibited reduced clinical severity, diminished epidermal thickening, and lower dermal leukocyte infiltration, along with a marked decrease in scratching behavior - an indicator of pruritus. Topical treatment with STA - 9090 also resulted in reduced serum IgE concentrations and restored epidermal barrier function, as evidenced by increased filaggrin expression in lesional skin. These findings not only support the involvement of Hsp90 in the immunopathogenesis of AD but also demonstrate, for the first time, the therapeutic potential of Hsp90 blockade *in vivo*. Together, these data underscore the relevance of targeting Hsp90 as a novel strategy for modulating both immune dysregulation and barrier dysfunction in AD (29).

Subsequent studies have employed both cellular and murine models to further delineate the immunomodulatory effects of Hsp90 inhibition in the context of AD. Using both *in vitro* and *in vivo* systems, the immunomodulatory potential of Hsp90 inhibition has been systematically explored. In cultured primary human keratinocytes stimulated with proinflammatory cytokine combinations (TNF/IFNγ or TNF/IL-4), pharmacological blockade of Hsp90 with the small-molecule inhibitor RGRN - 305 led to a substantial downregulation of genes involved in both Th1 and Th2 immune responses, including proinflammatory cytokines (TNF, IL1B, IL6) and chemokines typically upregulated in AD (CCL17, CCL22, TSLP). In parallel, application of RGRN - 305 in a murine model of AD induced by topical MC903 not only alleviated visible skin symptoms such as erythema and edema, but also significantly reduced leukocyte infiltration within the dermis, including T cells, mast cells, and neutrophils. Notably, the therapeutic efficacy of topical RGRN - 305 was comparable to that of topical corticosteroids, such as dexamethasone, yet was associated with fewer systemic effects, including minimal weight loss. Transcriptomic profiling of inflamed skin revealed that treatment with RGRN - 305 reversed the disease-associated gene expression signature, particularly genes linked to immune activation and cytokine signaling (Il1b, Il4, Il6, Il13). Mechanistic investigations further demonstrated that Hsp90 inhibition interfered with intracellular signal transduction by reducing activation of STAT3 and STAT6 - transcription factors critically involved in cytokine-driven inflammation—thus providing a plausible molecular basis for the observed anti-inflammatory effects. Taken together, these results underscore the central involvement of Hsp90 in AD pathogenesis and support the concept that its inhibition - especially through agents like RGRN - 305 - may represent a novel therapeutic strategy capable of modulating key inflammatory and immune pathways in AD (30).

Adding a crucial piece to the puzzle, Sitko et al. (2025) provided a comprehensive analysis of the effects of topical 17-AAG, a well-characterized Hsp90 inhibitor, in the DNCB-induced murine AD model, alongside translational data from human AD patients. The study showed significant clinical improvement in treated mice, evidenced by reduced dermatitis scores. Histologically, 17-AAG treatment correlated with reduced epidermal hyperplasia and decreased expression of TSLP, IL - 5, and IL - 6 in skin biopsies. Importantly, while the number of infiltrating leukocytes - including eosinophils, mast cells, and CD4+ T cells - was not significantly altered by treatment, the activity of eosinophils and mast cells was suppressed, as indicated by significantly reduced eosinophil peroxidase (EPX) activity and lowered serum histamine levels in 17-AAG-treated AD mice. Molecular analyses demonstrated effective downregulation of proinflammatory cytokines and inhibition of NF-κB activation in the skin. *In vitro*, 17-AAG inhibited the proliferation and inflammatory cytokine production of activated human keratinocytes and CD4+ T lymphocytes, including suppression of IL - 5, IL - 17A, and IL - 22, characteristic of Th2, Th17, and Th22 responses. Furthermore, 17-AAG dose-dependently reduced reactive oxygen species (ROS) production in activated human eosinophils, indicating decreased eosinophil activation status. Microbiome analysis by next-generation sequencing revealed that topical 17-AAG partially restored the gut and skin microbial balance disrupted in AD mice. Although the overall abundance of *Staphylococcus aureus* on the skin was not significantly changed *in vivo*, 17-AAG potently inhibited *S. aureus* biofilm formation *in vitro* in a dose-dependent manner, suggesting an additional mechanism contributing to the clinical benefit. Importantly, analysis of tape-strip samples from human AD patients revealed no significant differences in the gene expression of four HSP90 homologs (HSP90AA, HSP90AB, Grp94/gp96, and TRAP1) between lesional and non-lesional skin. Similarly, total HSP90 protein levels in polymorphonuclear leukocytes (PMNs) and peripheral blood mononuclear cells (PBMCs) did not differ significantly between AD patients and healthy individuals. However, Sitko et al. observed a significantly lower level of acetylated HSP90 (acetyl-HSP90) in PBMCs from AD patients compared to controls, while treatment of human keratinocytes (HaCaT cells) with 17-AAG led to increased acetylation of HSP90. Additionally, elevated eosinophil peroxidase (EPX) activity was detected in leukocytes from AD patients, consistent with increased eosinophil activation in the disease. These findings highlight that the pathological role of Hsp90 in AD is not due to its expression levels but rather its functional state and post-translational modifications such as acetylation. Together, the data support the potential of topical Hsp90 inhibitors like 17-AAG to modulate immune activation, restore barrier function, and normalize the microbiome in AD, offering a promising therapeutic avenue (31).

These translational findings align with mechanistic insights into the role of Hsp90 in contact hypersensitivity, a model immunologically related to AD. Kim et al. (2022) demonstrated that both pharmacological inhibition and shRNA-mediated knockdown of Hsp90 markedly suppressed DNCB-induced immune activation *in vitro*. Moreover, genetic deletion of Hsp90 significantly attenuated contact hypersensitivity responses in mice. Additional experiments suggested that DNCB-modified Hsp90 activates immune responses via the CD91 receptor, as CD91 blockade in cell lines and *in vivo* led to comparable reductions in hypersensitivity (32).

**Figure 1:**
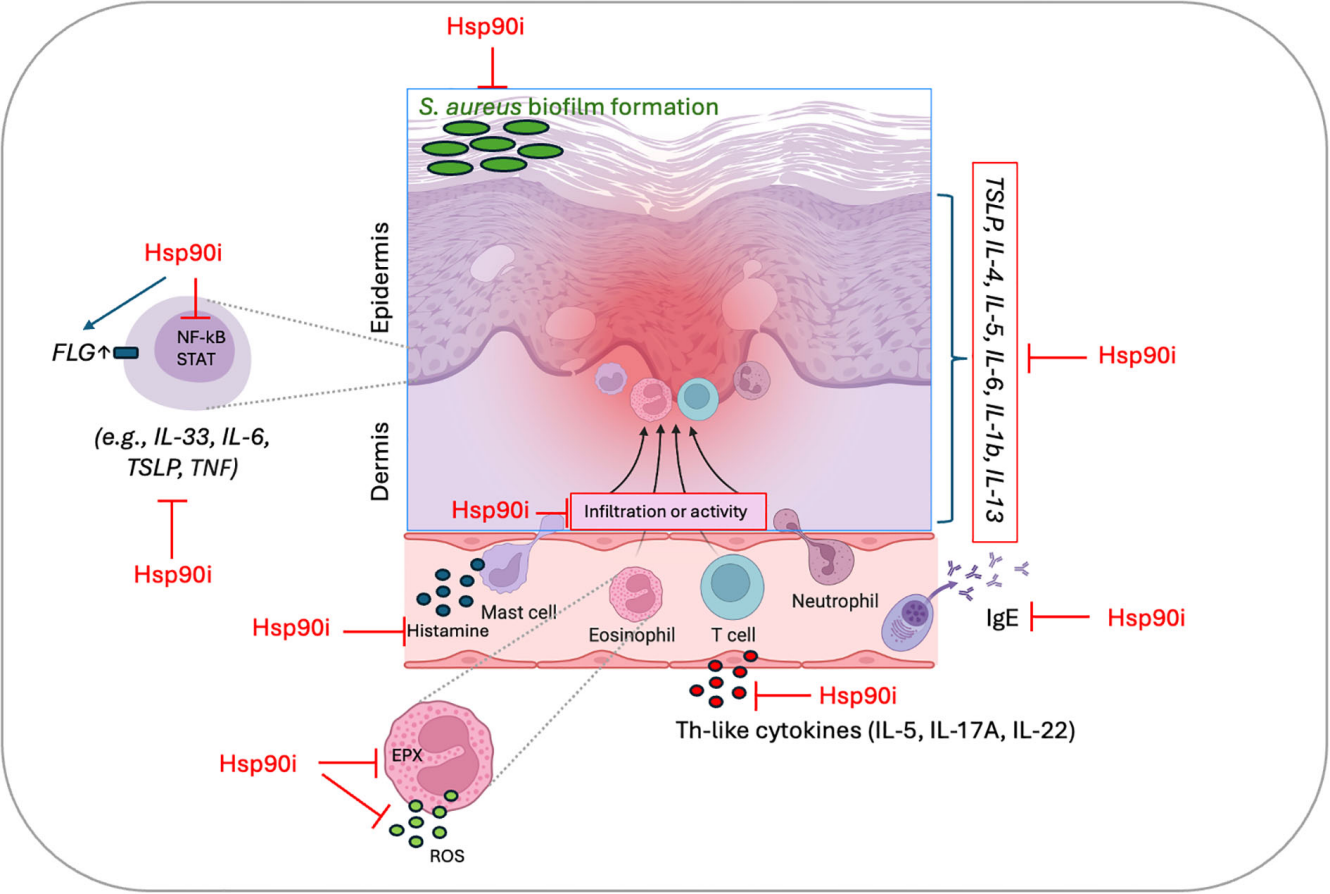
Proposed pathophysiological mechanisms and therapeutic targets in preclinical models of atopic dermatitis (AD) modulated by Hsp90 inhibition. Hsp90 inhibition affects multiple pathways, including suppression of proinflammatory cytokine and IgE production, inhibition of histamine release, attenuation of NF-κB/STAT signaling and EPX activity, reduction of ROS generation, inhibition of dermal leukocyte infiltration, and restoration of epidermal barrier integrity through FLG upregulation. The diagram summarizes current evidence described in references (29–32). Hsp90i, heat shock protein 90 inhibitor; EPX, eosinophil peroxidase; ROS, reactive oxygen species; FLG, filaggrin; NF-κB, nuclear factor kappa-light-chain-enhancer of activated B cells; STATs, signal transducer and activator of transcription proteins; TSLP, thymic stromal lymphopoietin; IL, interleukin; Th, T helper cells; IgE, immunoglobulin E.

Together, these studies underscore Hsp90 as a nodal regulator of key pathological processes in AD, including keratinocyte activation, T cell differentiation, cytokine production, epidermal barrier integrity, and skin microbiome composition. The consistent therapeutic benefits observed with diverse Hsp90 inhibitors, such as Geldanamycin, Ganetespib, 17-AAG, and RGRN - 305, administered systemically or topically, provide strong rationale for further clinical evaluation in patients with AD (**Table 1**). The proposed pathophysiological mechanisms and therapeutic targets in preclinical models of AD modulated by Hsp90 inhibition are also presented in **Figure 1**.

Notably, while extracellular Hsp90α is significantly elevated in the serum of patients with AD (27), corresponding increases in total Hsp90 protein within lesional skin are not consistently observed (31). This suggests compartment-specific regulation of Hsp90 in AD. Extracellular Hsp90 may be actively secreted or released into circulation in response to systemic inflammation or skin barrier impairment, where it can function as a danger-associated molecular pattern (DAMP) and modulate immune responses. In contrast, intracellular Hsp90 expression in keratinocytes and other skin-resident cells appears to remain relatively stable, possibly due to homeostatic regulation. Moreover, functional modifications of Hsp90, such as acetylation, rather than changes in overall protein abundance, may critically influence its activity in AD pathogenesis. These post-translational modifications can alter Hsp90’s chaperone function and interactions with client proteins, thereby affecting inflammatory signaling without necessitating altered expression levels in the skin. This compartmentalized and functional regulation of Hsp90 underscores the complexity of its role in AD and supports the rationale for targeted therapeutic modulation (31).

In conclusion, the accumulated evidence supports Hsp90 as a promising therapeutic target in AD. Its inhibition exerts multi-level effects on inflammation, epidermal barrier function, and skin microbiota, all critical components of AD pathogenesis. Notably, recent findings indicate that the four Hsp90 isoforms - Hsp90α, Hsp90β, GRP94, and TRAP1- may exert distinct and even opposing effects on inflammatory pathways in the skin. Selective inhibition of TRAP1 has been shown to consistently suppress proinflammatory cytokine expression in keratinocytes, fibroblasts, and lesional tissue from patients with hidradenitis suppurativa (33). Although such data are not yet available in the context of atopic dermatitis, this raises the possibility that isoform-specific targeting – particularly of TRAP1 – could offer a more refined therapeutic approach. Future studies will be required to determine whether similar mechanisms operate in AD, and whether TRAP1 inhibition might prove beneficial in this specific disease context.

## Perspective

Recent clinical investigations of the oral Hsp90 inhibitor RGRN - 305 in plaque-type psoriasis and hidradenitis suppurativa (HS) have provided promising evidence supporting the therapeutic relevance of Hsp90 inhibition in chronic inflammatory skin disorders (11, 34). In an open-label proof-of-concept trial, treatment with RGRN - 305 led to marked clinical improvement in a subset of patients with moderate-to-severe psoriasis, accompanied by molecular and histological normalization of key inflammatory pathways, including TNF-α, IL - 23, and IL - 17A signaling (11). Similarly, in a double-blind randomized trial in HS, RGRN - 305 demonstrated clinically meaningful efficacy and a favorable safety profile, with over half of treated patients achieving HiSCR-50 responses and no serious adverse events reported (34). These findings highlight the potential of Hsp90 as a viable therapeutic target beyond oncological indications (**Table 1**).

Although Hsp90 inhibitors have not yet been clinically evaluated in patients with AD, existing data suggest a manageable safety profile that may translate to this population. In the psoriasis study, four of seven patients receiving 500 mg/day of RGRN - 305 developed a mild-to-moderate exanthematous drug-induced eruption, leading two to discontinue treatment (11). Importantly, no severe adverse events were reported, and treatment-emergent events were comparable in frequency between RGRN - 305 and placebo groups in the HS trial (34). While systemic administration at higher doses may pose tolerability challenges, these findings underscore the importance of optimizing dosing strategies. For AD, where barrier function is compromised and long-term safety is paramount, lower systemic doses or localized (e.g., topical) delivery of Hsp90 inhibitors could offer a more targeted and better-tolerated approach. Future studies in AD populations will be critical to determine the risk–benefit profile in this context.

Given the mechanistic overlap in cytokine signaling and barrier dysfunction across psoriasis, HS, and AD, Hsp90 inhibition may hold promise in AD, where conventional therapies often fall short in patients with severe, chronic, or treatment-resistant disease. Notably, preclinical data - including those discussed in this mini-review - indicate that Hsp90 contributes to AD pathophysiology at multiple levels: from modulating T cell differentiation and cytokine production to regulating keratinocyte stress responses and skin–microbiota interactions. In parallel, recent studies have shed light on the role of Hsp90 in AIBDs, such as epidermolysis bullosa acquisita and bullous pemphigoid (12). Collectively, these studies provide converging evidence that Hsp90 plays critical roles in AIBD pathogenesis by supporting pathogenic autoantibody production, neutrophil effector functions, and keratinocyte inflammatory responses. Pharmacological inhibition of Hsp90 has been shown to modulate disease-driving mechanisms at multiple levels, offering a promising therapeutic strategy with potential for both systemic and topical application. The molecular chaperone Hsp90 has thus emerged as a key regulator of inflammatory and autoimmune mechanisms in dermatology. Taken together, the emerging clinical efficacy of Hsp90 inhibitors in related dermatoses, coupled with robust preclinical support in AD and AIBD models, strongly supports the initiation of early-phase clinical trials in patients with AD. Such studies may pave the way for a new class of host-directed, immunomodulatory therapies targeting Hsp90 in inflammatory skin disease.
